# Why Chaplaincy at Asylum Centers is a Good Idea: A Care Ethics Perspective on Spiritual Care for Refugees

**DOI:** 10.1007/s10943-023-01889-2

**Published:** 2023-08-26

**Authors:** Pieter Dronkers, Joanna Wojtkowiak, Geert Smid

**Affiliations:** 1 Department of Care Ethics, University of Humanistic Studies, Utrecht, The Netherlands; 2 Department of Humanist Chaplaincy Studies, University of Humanistic Studies, Utrecht, The Netherlands; 3 ARQ Centrum’45 / ARQ National Psychotrauma Centre, Diemen, The Netherlands

**Keywords:** Asylum seekers, Refugees, Chaplaincy, Spiritual care, Care ethics, Multicultural theory, Asylum centers

## Abstract

This article argues in favor of introducing chaplaincy care at asylum centers and develops three arguments for doing so. First, chaplaincy is one way to protect the right to health of refugees and to improve their spiritual well-being. The positive contribution of chaplaincy services to mental health care is increasingly recognized, especially in the domain of PTSD. Second, chaplaincy services support asylum seekers in exercising their freedom of religion while entrusted to state care. Chaplains can create a safe space for asylum seekers to reflect on their spiritual and religious needs, orientation and belonging. Third, chaplains are well positioned to help asylum seekers in rebuilding their life-sustaining web, while at the same time promoting a climate of inclusion and respect in and outside the asylum center.

## Introduction

Recent studies indicate that spirituality can be a valuable resource for positive coping and the mental health of asylum seekers who have fled their home country (Maier et al., 2022; Pandya, 2018).^1^ A quantitative study from Germany among 744 refugees showed that attending to the spiritual needs of refugees can positively contribute to their well-being. It helps them cope with the uncertain situation they are in (Maier et al., 2022). Offering space for spiritual care is also important as many refugees come from countries where religion plays a key role in daily life. Therefore, in some Western destination countries, (pilot) programs have been setup to include spiritual education programs and chaplaincy services in the mental health care arrangements for refugees (Kimball et al., 2022; Schmid & Sheikhzadegan, 2020). Although the success of such initiatives indicates the urgency of spiritual care, they also provoke questions about the expediency of such (sometimes faith-based) chaplaincy services in secular contexts (Lassen, 2019, p. 60).

We will argue that chaplains who are trained as nondenominational spiritual care professionals and who at the same time can offer faith-based spiritual care have a meaningful and significant contribution to make to the spiritual well-being of residents. Spiritual well-being refers to “the core of a person” (Chirico, 2016, p. 14) and comprises “cognitive and behavioral efforts to find or maintain meaning, purpose and connection in the face of difficult situations” (Coppola et al., 2021, p. 2). Spirituality is “a source of comfort and meaning”, offers “a sense of belonging and existential interconnectedness” (Coppola et al., 2021, p. 2) and has been linked to resilience. We believe that the knowledge and interventions of faith-based chaplains are vital as well, though appointing them might lead to questions in a secular or interfaith context.

The structure of this paper is as follows. After an introduction describing our normative framework and chaplaincy care, we first consider the responsibility of destination countries to secure the right to health of refugees and elaborate on chaplaincy care as an intervention that contributes to mental and spiritual well-being. Secondly, we discuss how the freedom of religion or belief is an additional justification and incentive to offer faith-based spiritual care. Thirdly, we argue that chaplaincy care is pivotal as it helps refugees in building connections inside the asylum centers as well as with the broader host society. Finally, we emphasize the urgency for societies to include refugees in their circle of care.

## Human Rights, Care Ethics and Spiritual Care

In our discussion of chaplaincy in asylum centers, we will combine a human rights perspective with care ethics. The former emphasizes the power of individuals to claim goods from the government that are crucial for their survival and flourishing. Human rights focuses on and protects the fundamental needs of individuals. Meanwhile, the starting point of care ethics is a relational ontology founded on the observation that people, as vulnerable beings, are mutually dependent for survival and good life (Engster & Hamington, 2015, pp. 3–4). Given this interdependence, care is a crucial activity “that includes everything that we do to maintain, continue, and repair our ‘world’ so that we can live in it as well as possible” (Fisher & Tronto, 1990, p. 40). To care is to weave “a complex, life-sustaining web that integrates our bodies, our selves, and our environment” (Tronto, 2013, p. 19). If human interdependence is indeed fundamental, then the key political questions to ask are whether the needs for care of all human beings are sufficiently recognized and addressed, as well as whether the responsibilities of care are equally distributed and democratically discussed (Tronto, 2013, p. 62). These typical care ethical concerns offer a context to analyze how citizens and governments care for refugees or fail to do so (Dronkers, 2022; Morgan, 2020; Nipperess, 2018).

While the discourse of human rights protects the fundamental needs of individuals, care ethics helps to recognize and reflect on the normative questions arising from the mutual interdependence of individual human beings, refugees included. Through care work, people are constantly weaving and repairing their life-sustaining web. The fundamental experience of people who have had to flee is that their network is torn apart, resulting in existential disorientation. Refugees must find their way between a home that has been lost, and a place that is not yet home (Ahmed, 2006, 10). The vocabulary of care ethics helps recognize and discuss the urgency of supporting people in repairing their interconnectedness, for instance through spiritual care, and to see that this is not only an individual task but a collective one as well.

## What can Chaplains Contribute?

Before we discuss the question of why asylum centers should offer chaplaincy, we first have to define this type of care itself. While doing so, we will mainly build on examples from the Netherlands, as in the Dutch context there are already different types of chaplaincy services in place that offer distinct models for how spiritual care at asylum centers could be organized.

The Association for Spiritual Caregivers in the Netherlands (VGVZ) describes spiritual care as “professional support, guidance, and consultancy  regarding meaning and world views” (2023, p. 4). According to VGVZ, the work of chaplains comprises three different domains. First, chaplains guide people, individually or in groups, in finding what makes them stronger and gives inspiration, in line with their biography and worldview. Rituals and sacraments can be part of this. Second, when people are dependent on care, are detained, or serve in the military, chaplains provide spiritual assistance as well as a safe space, and thus help individuals to exercise their constitutionally enshrined right to freedom of religion or belief. Third, chaplains support their colleagues, advise management in the field of spirituality and ethics, provide training for, and contribute to an inclusive and respectful climate concerning religion and worldview.

The field of chaplaincy is developing quickly. Next to traditional faith-based forms, a nondenominational model of chaplaincy has gained ground (Jacobs et al., 2021, pp. 85–87). In this latter approach, the tasks of chaplains are no longer primarily related to representing specific worldviews, but center around providing nondenominational spiritual care to anyone in need of professional guidance and counseling. The professional identity of chaplains has “gradually shifted from a religiosity-based profession to a broader profession providing caring for all existential needs, away from institutional care provision to more individual spiritual support” (Glasner et al., 2023, p. 132). In a recent scoping review, the following goals of chaplaincy have been identified: promoting worldview vitality and well-being, processing life events, deepening spirituality, exercising freedom of religion, and relational affirmation (Visser et al., 2023). Dutch spiritual care scholars Schuhmann and Damen (2018, p. 408) defined the role of chaplains as follows: “[they] meet with people in situations that may be understood in terms of disorientation in moral space. People who face loss, illness, death, incarceration﻿, or violence may feel that their sense of being close to or moving towards ‘the good’—towards a life worth living or a full life—is challenged or even shattered.” Chaplains, thus, accompany people in moments of disorientation, such as (forced) migration; they guide their search for the good in a world that is at times unfair and hurtful. They can offer a qualified conversation about meaning in life, moral dilemmas, hopes and fears, guilt, and loss to those who are facing and have faced severe adversities in life. What “a life worth living” means is not presented by the chaplain but arises in the dialog between the chaplain and client.

As sociologist Nancy Ammerman (2010) argued, in increasingly diverse societies, people have access to and are building on a variety of sources for meaning-making, some of them religious, some of them personal, social or cultural, and anything in between. For refugees specifically, religiosity can be a source of meaning *and* disorientation (Adedoyin et al., 2016; Fensham, 2021). Spiritual and religious beliefs and practices have been found to be positively related to the well-being of refugees and a strategy to deal with the challenges and for instance, discrimination experienced in host countries (Adedoyin et al., 2016; Hodge, 2019). Religiously involved LGBTQIA + refugees, however, for instance, have reported on “experiences of internalised religious messaging inducing shame, self-blame, and suicidal ideation and attempts at suicide which led to a rejection of organised religion.” (Fensham, 2021, p 4). What is more, LGBTQIA + refugees do experience discrimination from the native population. Chaplains can create room to discuss spiritual and religious concerns in relation to one’s identity, gender and sexual orientation, as well as make room for expressing one’s cultural and religious identities.

To ensure the space for meaningful encounters and conversations, chaplains often operate on the border of the “religious” and the “secular.” Research on interfaith chaplaincy from the US shows that spiritual caregivers developed various strategies to engage with clients from different backgrounds. Sometimes they use neutral language to address existential and spiritual questions of those clients who do not identify with a specific worldview, and on other occasions, they code-switch between the worldview of the client and their own beliefs more explicitly, and translate them so that the client can relate their own narrative and point of view to them (Cadge & Sigalow, 2013). During the COVID-19 pandemic, for instance, chaplains proved on a large scale that they can offer spiritual care to various groups of worldviews and that they can improvise in moments of crisis (Flynn, Tan & Vandenhoek, 2021).

Chaplains have a range of interventions at their disposal to address the existential, spiritual, and religious needs they encounter. Handzo et al. (2008) studied the actions chaplains reported on in health-care settings in the New York area in the US. The researchers divided interventions into “specifically religious” (e.g., prayer, blessing, or confession) and “general activities,” such as life review, empathic listening, or ethical consultation (Handzo et al., 2008, p. 43). The most common religious intervention was prayer, mostly among Muslim (77%), Roman Catholic (59%), Protestant (58%) and also among 46% of non-affiliated patients (Handzo et al., 2008). Moreover, Jewish and Muslim patients were supplied (both 16%) with religious items, such as Sabbath candles, a Qur’an, or prayer rugs. Most reported general interventions were empathic listening (in 72% of chaplain visits), emotional enabling (15%) and life review (12%). We can conclude that chaplains use interventions that focus more on psycho-emotional aspects as well as interventions that focus more on spiritual-religious aspects of meaning-making (and some that can address both). Some existential issues can be addressed by all chaplains, but it must also be noted that different groups of clients have specific religious needs that can “only” be served by faith-based chaplains.

In the Dutch context, both faith-based and nondenominational forms of chaplaincy are well-established. Faith-based chaplains generally have a religious endorsement or mission and are appointed in a representative way to ensure that the available chaplains encompass all the relevant religious worldviews. Often, these chaplains will be familiar with the language and cultural background of their clients and sometimes have their own personal experiences with migration (Schmid & Sheikhzadegan, 2020, p. 128). An increasing number of chaplains work from an interfaith or nondenominational perspective, which means that they address existential and spiritual issues and needs of clients but do not explicitly identify with one specific religious denomination. These “territorial”﻿ spiritual care givers are often recruited and appointed directly by employers, like nursing homes and the police. As individuals, nondenominational chaplains can have a religious, or a humanist, background, but the recognition and evaluation of their work is increasingly dependent on evidence-based efficacy and professional standards, rather than on the protection of the freedom of citizens to adhere to a specific religion or worldview. As we substantiate in the next sections, these two different types of chaplaincy are also related to distinct justifications for offering spiritual care at asylum centers.

## Chaplaincy as Spiritual Guidance and the Right to Health

The first argument for introducing chaplaincy in asylum centers is that, as an increasingly recognized mental health intervention, it is one way of fulfilling the obligation of states to protect the right to (mental) health of refugees. The 1966 International Covenant on Economic, Social and Cultural Rights (ICESCR) stipulates that each individual should be able to enjoy the highest attainable standard of physical and mental health (Derckx, 2017, pp. 124–125).^2^ State parties to this treaty have the obligation to respect, protect, and fulfill this right, for instance through ensuring access to health facilities, goods and services on a nondiscriminatory basis, especially for marginalized groups.^3^ As the UN Committee on Economic, Social and Cultural Rights (UN CESCR) emphasized in 2017, these groups include refugees, asylum seekers, and undocumented migrants.^4^

Access to mental health care is indeed crucial for these newcomers, as research shows that they have increased rates of short- and long-term mental health problems. These are related to the situations they fled from and to the hardships they experienced during their flight (Koesters et al., 2018, p. 369). PTSD is among the most frequently reported mental health problems. In a meta-analysis of studies among refugees and other conflict-affected persons, mean prevalence rates of 30.6% were found (Steel et al., 2009). Even after their arrival in a host country, asylum seekers remain vulnerable to the development of PTSD, especially when they are exposed to additional stressors, such as delays in the processing of their applications, difficulties in dealing with immigrant officials, obstacles to employment, loneliness, boredom, and losses of loved ones (Hengst et al., 2018). Many asylum seekers need access to individual therapy and other specialized interventions. Having a chaplain present in asylum centers would give residents the opportunity to freely discuss their concerns and worries, their existential and spiritual suffering and sense of disorientation. It would bring a positive presence within the long and bureaucratic process of waiting for asylum. When promoting spiritual well-being relates to more resilience in other situations, such as during the COVID-19 pandemic (Coppola et al., 2021), it should be expected to help in this context as well.

Moreover, it is specifically in the domain of preventing or recovering from PTSD that there is a growing recognition of the positive contribution that spiritual care can make, as it creates space for individuals to reflect on their existential and spiritual processes and biography (Schuhmann & Damen, 2018, p. 414). Chaplaincy contributes to health as the “capacity to cope and maintain and restore one’s integrity, equilibrium, and sense of well-being” (Huber et al., 2011). Of the five current international guidelines on PTSD (Hamblen et al., 2019), two explicitly recommend chaplaincy, pastoral, existential, or spiritual care for various trauma-exposed populations, including the military (Department of Veteran Affairs & Department of Defense, 2017; Phoenix Australia Centre for Posttraumatic Mental Health, 2013), and communities following large-scale disasters (Phoenix Australia Centre for Posttraumatic Mental Health, 2013). Two other guidelines emphasize the need for research on the efficacy of complementary existential, spiritual, and meaning-making interventions (American Psychological Association, 2017; International Society for Traumatic Stress Studies, 2018). As these guidelines suggest, chaplaincy can be fruitful in supporting refugees in working towards spiritual and mental well-being, especially while refugees are still in the uncertain position of seeking asylum and waiting for a decision about their application.

Chaplains can also positively contribute to the health of refugees by helping to facilitate access to other health care services, thus furthering equity in health care access. Especially when they do share—or understand—the worldview and native language of asylum seekers, chaplains, as persons of trust, with dialogical skills (Jacobs et al., 2021, p. 91), can achieve this in three primary ways.

First, asylum seekers often experience *practical* difficulties in finding their way within a health-care system that is new to them (Giacco & Priebe, 2018, p. 111). Chaplains can help to tailor the relevant information, specifically to individuals with lower levels of literacy (cf. van Loenen et al., 2018, 85). Second, even when newcomers know their way around in the system, they might not *trust* the doctors or the health-care institutions. Different cultural codes can easily create misunderstandings and thus hamper the development of trust (Shors & Kroll, 2022, p. 247). Here too, chaplains could play an intermediary role, specifically in medical cases of shared decision-making, as they are likely to have a proper understanding of relevant values and worldviews held by both the refugees and the care professionals (cf. Laverack, 2018). Third, the way in which refugees recognize, frame, and experience mental disorders can differ from how these are interpreted in the host society (Satinsky et al., 2019, pp. 861–862). Asylum seekers may understand mental disorders as mere “craziness,” or as a spiritual, social, or moral challenge (Weissbecker et al., 2018). Chaplains who are familiar with different worldviews and cultural horizons can contribute to counteracting *stigmas,* and thus make it easier for people to recognize that they might need specialized care and that this is not a shameful thing (Satinsky et al., 2019, p. 861).

## Chaplaincy as Safe Space and the Freedom of Religion or Belief

Providing chaplaincy care is also critical as it enables refugees to exercise their freedom of religion or belief. The 1951 Refugee Convention states in article 4: “Contracting States shall accord to refugees within their territories treatment at least as favorable as that accorded to their nationals with respect to the freedom to practice their religion.” This implies, first of all, that states should refrain from interfering with individual beliefs and practices related to religion or worldview, as long as these do not disturb the public order. However, this freedom also entails that, in specific cases, states are under the positive obligation to ensure that individuals can actually enjoy their rights. Organizing chaplaincy care is one of the ways to fulfill this responsibility (Lassen, 2019, p. 54).

Under which circumstance does such a positive obligation exist? When refugees are held in detention, for instance, while awaiting their deportation, it is clear that they should be given access to spiritual or pastoral care and counseling (Association for the Prevention of Torture & UNHCR, 2014, pp. 154–158). When they are staying in an asylum center and are free to leave the premises, then the obligation to provide chaplaincy is less obvious. However, asylum seekers might not be able to fully enjoy their freedom of religion in the mixed public/private context of the asylum center either. While residents must check in with authorities at the center on a regular basis, relevant religious communities might be at a large geographical distance, prayer rooms might be lacking, dietary rules and other ritual prescriptions might be difficult to follow. What actions asylum centers should and could take to actually protect the freedom of religion or belief and remain neutral institutions at the same time is an understudied topic (Lassen, 2019, p. 60). Due to the focus of this article, we will limit our discussion here to chaplaincy.

Why would states be under any positive obligation to accommodate the spiritual needs of their residents, especially in increasingly secularized destination countries such as the Netherlands? Proponents of accommodation argue that culture—including religious traditions—matters, as part of the way we give meaning to the world, and as an essential element in our self-ascribed identity (Phillips, 2007, p. 15). Individuals develop their sense of self through the relationships, organizations, and communities they are part of (Parekh, 2008, p. 15). This implies that the possibility to live a good life is intimately entwined with access to, the flourishing of, and respect for cultural practices and traditions that people hold dear (Kymlicka, 2007, p. 30). Although liberal democracies and their institutions should remain neutral vis-à-vis the worldviews of their inhabitants, protecting the conditions for a good life can be a legitimate reason for states to support citizens in accessing and practicing cultural and religious traditions that are meaningful to them. This might be especially vital for refugees who often come from less-secularized societies. Here, it should be emphasized that accommodation policies can only be justified if all communities receive the same treatment, which political philosopher Joseph Carens (2000) labeled the precondition of even-handedness.

Opponents of this type of active state engagement emphasize that accommodation can come at the expense of the freedom of individuals and minorities within minorities, such as women, LGBTQIA + -people, or individuals with deviating political opinions (Phillips, 2007, p. 12). The religious and cultural organizations with which the government has an official connection, for instance for recruiting faith-based chaplains, see their social status and regulatory power increased at the expense of different voices (Phillips, 2007, p. 169). For some liberal feminists, these are reasons to be skeptical about any type of accommodation. In their view, states should refrain from cooperating with and supporting cultural and religious organizations and the services they provide (Shachar, 2007, pp. 117–123).

Recognizing both the importance of cultural and religious traditions for individual identity, as well as the extent to which specific practices and convictions can be harmful, political theorist Anne Phillips (2007) argued for accommodation arrangements that take as their starting point the protection of the rights of individuals rather than those of communities. The aim of such policies is to protect and support individual agency in the context of increasing religious and cultural diversity. Phillips defines agency “as the capacity to reflect on and, within the limits of our circumstances, either endorse or change the way we act or live–thus, in some significant sense, to make our actions and choices our own” (Phillips, 2007, p. 101). While our beliefs, values and choices are pervaded by cultural assumptions and norms, we are at the same time actors who produce, embrace, contest, and reinterpret these cultural practices and traditions (Phillips, 2007, p. 45). Protecting and supporting individual agency requires both acknowledging the crucial role of culture and worldviews for giving meaning to our lives, and simultaneously recognizing the need to have the freedom to make individual choices amid the complex interactions with the structures in which these traditions are embedded.

Phillips’ interpretation of agency as the capacity to endorse or change the way we act and live helps to see how the freedom of religion protects the safe space for this dynamic and ongoing process of living and reviewing our beliefs (Nagy & Speelman, 2017, p. 359). And it is precisely here that chaplains can support asylum seekers, as they provide them with the time and space to reflect on their biography and to find a sense of orientation during a highly stressful period in their lives.

Chaplains who share the religious beliefs of refugees can assist in exploring existential religious struggles, or in answering practical questions related to practicing their faith in a new context. They can also support people in reconnecting to the rituals, feasts, and communal activities that have always been dear to them. In this way, refugees can restore the link with the home they have lost (Mayer, 2007, p. 7). At the same time, it is equally important that people also have the opportunity to consult nondenominational chaplains. These professionals can offer space for those who want to redefine their relation to, or even break away from, cultural or religious traditions they grew up with. Such chaplains also provide guidance in finding individual ways to (ritually) express grief, integrate loss, and explore new beginnings.

In short, chaplaincy care at asylum centers helps refugees to exercise their freedom of religion and offers space for finding orientation in a complex period of their lives. Protecting individual agency requires that refugees can opt for either faith-based, or nondenominational chaplaincy services. To counter anxieties about religious oppression or radicalization, all chaplains should be well trained in dialog, dealing with religious diversity and differing worldviews. The willingness to cooperate with peers and to be held accountable are essential competencies to guarantee chaplaincy care that promotes individual agency. Such professional standards are already required and successfully applied in other types of institutional chaplaincy (Vellenga & de Groot, 2019, p. 233).

## Chaplaincy Contributing to a Climate of Inclusion and Respect

The third reason to introduce chaplains is that they are trained to facilitate an inclusive and respectful climate that offers space for religious and philosophical diversity (VGVZ, 2023, p. 4). The right to health and the freedom of religion protects the position of individuals over and against collectives that can be neglectful, indifferent, or even oppressive. A care ethics perspective is relevant to discuss how people are embedded in and dependent on supportive networks but on which people cannot lay any legal claim. Tronto’s (2013, p. 31) concept of a “life-sustaining web” helps to recognize the normative importance of this dimension of interrelatedness: “All individuals constantly work in, through or away from relationships with others, who in turn are in differing states of providing or needing care from them.” Humans are relational beings, and it is therefore important to provide them with support in building—and repairing—their life-sustaining networks. There are three ways in which chaplaincy care can do this.

First, chaplains can play a role in securing a supportive climate of inclusivity and respect at the asylum centers themselves through advising management, training staff members, and offering educational programs (cf. Jacobs et al., 2021, p. 92). The temporary cohabitation of residents can be complex and tense, especially when people come from different religious and cultural backgrounds. In a context in which social safety is often under pressure, it is crucial to invest in preventing tensions and misunderstandings between the different groups.^5^ This could be done by adopting a strategy of *active pluralism*, which entails organizing engagement with others’ traditions, for instance during interfaith encounters and dialog programs focused on tolerance and mutual understanding (Dalsgaard, 2019, p. 34; De Hert & Meerschaut, 2007, p. 6). In 2017, the Central Agency for the Reception of Asylum Seekers (COA) in the Netherlands, together with several interreligious platforms, setup a successful encounter-based program on preventing discrimination on any ground, including religion and sexual orientation.^6^ Experiences in the health-care sector show that chaplains trained in interfaith work are well positioned to help in overcoming the negative impact of religious divisions (Cadge & Sigalow, 2013, p. 156).

Second, chaplains can assist asylum seekers in building a network outside the asylum center. They can put them in touch with people with similar existential questions, or with relevant organizations and support groups. Chaplains who share the religious background of the newcomers are in a good position to link people to relevant religious and diaspora communities (Dalsgaard, 2019, p. 29). Here too, it is crucial that individual agency is respected. Indeed, refugees might have good reasons to be hesitant in engaging and identifying with the people who left or fled the same country before them (Petersen & Jensen, 2019, p. 13). Divergent religious, cultural or geographical identities, social position, political affiliation, or fear for the long arm of the secret services can be among the motives for such reluctance. However, as long as it is on the asylum seekers’ own initiative, chaplains can support them in finding orientation and making new connections.

Third, chaplains may also contribute to a climate of inclusion and respect in the broader society. Especially in times of large influx, the hosting of refugees can result in societal polarization. In 2015, warning against the “immigration threat” became a common theme among populist politicians all over Europe. Religion, and in particular Islam, was used as a catalyst to fan anxieties about migration (Wilson & Mavelli, 2019, p. 19). At the local level, decisions to establish asylum facilities were highly controversial, causing moral panic and tense public debates. While refugees went through the actual and physical loss of home, citizens feared that they would have the same experience while staying in their own locales (Duyvendak, 2011, p. 23). Chaplains can play a constructive role in public debates about these anxieties. They can do so by explaining the plight of the newcomers, as well as their cultural and religious heritage, and thus contribute to the “humanization” of and a renewed solidarity with refugees (cf. Wilson & Mavelli, 2019, p. 24).

Inclusion and respect are complex concepts that are hard to articulate in the discourse of individual claim-making that is central to the human rights discourse. The recognition of our mutual dependence and the need to maintain our life-sustaining webs, however, helps justify why governments should invest in interventions that support these ideals. Spiritual care is definitely not the only discipline that has something to contribute here, but as specialists in dealing with different worldviews, ethical questions, and spiritual guidance, chaplains have a key role to play in furthering inclusion and respect.

## Inclusion in the Circle of Care

Throughout this article, the contribution of chaplains to the care for asylum seekers has been discussed from the perspective of human rights and care ethics. Chaplains can have at least three roles: 1) They provide guidance, orientation and space for reflection to asylum seekers and thus contribute to their spiritual and mental well-being; 2) They offer spiritual assistance and help individuals to exercise their freedom of religion or belief; and 3) They are relation-builders, ensuring a climate of inclusion and respect, while contributing to the refugees’ life-sustaining webs.

Investing in the spiritual and mental health of asylum seekers is urgent, as the right to health of this group is under severe pressure (Derckx, 2017, p. 124). In a broad European review, Puchner et al. (﻿^2018^) demonstrated that none of the signatories of the ICESCR fully meets the standards set by the Covenant. This leaves asylum seekers at risk of poor health and the general deprivation of basic rights (Bilecen, 2019, p. 43). After the memories of war and transit, the most reported drivers of mental health problems of newcomers are marginalization, hostility, and discrimination in the host country (Cornish et al., 2019, p. 168). Poor access to (mental) health care adds to the precariousness of the situation that asylum seekers already find themselves in (Derckx, 2017, p. 123).

From a care ethics perspective, the problem is not only that states fail to fulfill international obligations that they voluntary took upon themselves; they also neglect their responsibility to identify the needs of asylum seekers in a participatory way, to make transparent decisions about whether and how these needs can be addressed, and finally, to analyze the extent to which people themselves recognize that their needs are met. This last step is crucial as it upholds the agency of individuals, even if they are dependent on care (Tronto, 2013, p. 23). Failure to recognize and address the needs of refugees reinforces the impression that migrants too often remain “outside the space of compassion and of the impulse to care” (Bauman, 2016, p. 35). Providing chaplaincy care is one way to acknowledge some of the specific needs that asylum seekers have, thus making them more fully part of “the circle of care” of host societies (Hamington, 2015, p. 285).

Weaving a supportive web for refugees should be a task not only for newcomers and the formal and informal care providers around them; it is, in fact, a challenge for the host society as a whole, as the arrival of refugees requires mutual adaptation and integration. Through analyzing how societies (fail to) care for them, asylum seekers together with chaplains could identify where societal and political structures impede rather than encourage human flourishing (cf. Faist, 2018, p. 416). These insights would be important building blocks for a joint societal effort to build new connections and thus contribute to an inclusive society in which both newcomers as well as other citizens feel at home.

## Conclusions

In this article, we argued that the right to health, the freedom of religion or belief, and a climate of inclusion and respect provide compelling reasons to facilitate chaplaincy care for asylum seekers. If provided by both religious and nondenominational spiritual care givers that are well trained and committed to dialog and diversity, chaplaincy uniquely contributes to the spiritual and mental health, religious freedom, and life-sustaining networks of asylum seekers in their country of arrival. Future implementation and evaluation of chaplaincy care for refugees will pave the way for further professional development in this domain.

## Footnotes

1 In this article, the terms asylum seekers and refugees will be used interchangeably, because some of the research and literature we refer to does not distinguish between asylum seekers (individuals who applied for asylum and are awaiting a decision) and refugees (individuals who are recognized as such and have received a residence permit). We focus on the situation in asylum facilities, where the majority of the inhabitants are asylum seekers.

2 The ICESCR is ratified by 171 countries, including the Netherlands.

3 UN CESCR (2000). *General Comment No. 14: The Right to the Highest Attainable Standard of Health (Art. 12)*. E/C.12/2000/4, paragraph 43 (a).

4 UN CESCR (2017). *Duties of States towards refugees and migrants under the International Covenant on Economic, Social and Cultural Rights*. E/C.12/2017/1, paragraph 12.

5 Van den Berg et al. (2021). *LHBTI’s, bekeerlingen en religieverlaters in de asielopvang*. WODC, 22.

6 COA, Memo Initiatieven Informatievoorziening Grondrechten, 16 January 2018. Via: https://www.coa.nl/sites/default/files/2020-01/17%20Memo%20Initiatieven%20COA%20Info%20Grondrechten%2016%20januari%202018.pdf.
